# Recent advances in diabetic kidney disease

**DOI:** 10.1186/s12916-021-02050-0

**Published:** 2021-08-17

**Authors:** Mohamad Hanouneh, Justin B. Echouffo Tcheugui, Bernard G. Jaar

**Affiliations:** 1 Department of Medicine, Johns Hopkins University School of Medicine, 5601 Loch Raven Boulevard, Suite 3 North, Baltimore, MD 21239 USA; 2 Nephrology Center of Maryland, Baltimore, MD USA; 3 Department of Epidemiology, Johns Hopkins Bloomberg School of Public Health, Baltimore, MD USA; 4 Welch Center for Prevention, Epidemiology, and Clinical Research, Baltimore, MD USA

## What is diabetic kidney disease and what do we know so far about its clinical presentation?

Diabetes mellitus is the leading cause of chronic kidney disease (CKD) in the USA and worldwide. An estimated 422 million adults are living with diabetes globally, and up to 40% of them may develop CKD during their lifetime [1]. Diabetic kidney disease (DKD) does not reflect a specific pathological phenotype. In fact, it can be diagnosed clinically based on the presence of persistent albuminuria, sustained reduction in the estimated glomerular filtration rate (eGFR), or both in patients with diabetes [2]. DKD is usually identified after five years of the diagnosis of type 1 diabetes, while it can be recognized at the time of diagnosis of type 2 diabetes. The presence of proliferative diabetic retinopathy typically correlates with ongoing DKD in patients with albuminuria. Even though a kidney biopsy can confirm the diagnosis of DKD, this procedure is usually considered when an alternative diagnosis suspected.

Albuminuria has more recently been classified into moderate (30 to 300 mg/g) or severe (> 300 mg/g). Nonetheless, any degree of albuminuria has been associated with an increased risk for CKD progression, end-stage kidney disease (ESKD), adverse cardiovascular disease outcomes, and mortality in patients with diabetes [3]. A reduced eGFR in diabetic patients has been observed in the absence of albuminuria; however, the progression of DKD appears to be slower in these individuals [3]. Furthermore, the combined presence of albuminuria and lower eGFR independently increases the risks for cardiovascular events and mortality in individuals with diabetes [3]. The Kidney Disease: Improving Global Outcomes (KDIGO) and the American Diabetes Association (ADA) guidelines recommend that all diabetic patients undergo annual screening by checking serum creatinine-based eGFR and urine tests to evaluate for albuminuria [2].

## What is unique about the challenges with DKD compared with other types of kidney disease?

Individuals with type 2 diabetes may develop DKD before a clear diagnosis of diabetes is established. This has the consequence of delaying the diagnosis and appropriate treatment of DKD. More recently, we have witnessed significant progress in the treatment options for slowing DKD, but no real advance in reversing DKD. To date, available therapies are targeting DKD progression. Furthermore, not all DKD patients are eligible for these therapies because of variable side effects such as hyperkalemia, acute kidney injury (AKI), and extent of the DKD. Indeed, because of safety concerns, many of these newer medications are not approved for patients with eGFR below 30 mL/min/1.73 m^2^.

## What is known about the causes of DKD?

Hyperaminoacidemia, glomerular hyperfiltration and hyperperfusion, and hyperglycemia are the major metabolic abnormalities that affect the kidneys and are associated with inflammation and eventually fibrosis in diabetic patients [4]. The classic sequence of events in the natural history of DKD is driven by hyperglycemia in conjunction with hypertension and is characterized by glomerular hyperfiltration progressing to albuminuria, and then leading to a decline in kidney function. One mechanistic hypothesis suggests that a decrease in distal delivery of sodium chloride to the macula densa results from an increased proximal tubular reabsorption of glucose via sodium–glucose cotransporter 2 leading to a decrease in tubulo-glomerular feedback. This results in dilation of the afferent arteriole and increased glomerular perfusion [5]. On the other hand, increased production of angiotensin II leads to vasoconstriction in the efferent arteriole. The net effect is an elevated intraglomerular pressure leading to glomerular hyperfiltration [5]. Additionally, systemic hypertension and obesity can also contribute to glomerular hyperfiltration via glomerular enlargement [4].

A number of other factors can play a significant role in the pathogenesis of DKD. These include, for example, oxidative stress. Activation of advanced glycation end-products (AGE) receptors, which are represented on multiple cell types in the kidneys, induces the production of numerous cytokines. Hyperglycemia causes increased formation of AGE and activates protein kinase C, resulting in decreased production of endothelial nitric oxide synthase and increased levels of the endothelin 1, Angiopoietins 2, and vascular endothelial growth factor. Furthermore, hyperglycemia, angiotensin II, and AGE can activate macrophages, which are rich in cytokines and tumor necrosis factor. The net effect of these different pathways leads to endothelial instability, increased vascular proliferation, renal hypertrophy, podocyte injury, tubular epithelial cell injury, and increased cytokine production [6].

The structural changes of DKD start with thickening of the glomerular basement membrane followed by mesangial matrix expansion and foot process effacement [7]. Segmental mesangiolysis and Kimmelstiel–Wilson nodules are signs of DKD progression [8]. Interstitial fibrosis and global sclerosis develop in later DKD stages.

## What are the general treatment options for DKD?

Intensive glycemic control is critical in the prevention of DKD in the early course of the disease. However, a number of studies have shown that intensive glucose control may not reduce the risk of CKD progression or cardiovascular mortality in advanced stages of DKD [9]. The KDIGO guidelines recommend a target HbA1c ranging from < 6.5 to < 8.0%, with the choice of an exact target guided by the extent of hypoglycemia risk in each patient [10].

For glycemic control, current guidelines suggest using both metformin and sodium-glucose cotransporter 2 inhibitors for patients with DKD and GFR > 30 ml/min per 1.73 m^2^ [10]. Glucagon-like peptide-1 receptor agonists can be added to manage hyperglycemia if needed [10]. Uncontrolled hypertension can worsen DKD and increase the risk of progression to ESKD. The KDIGO guidelines recommend using an angiotensin-converting enzyme inhibitor (ACEi) or an angiotensin receptor blocker (ARB) to maintain blood pressure below 130/80 mmHg in all patients with CKD and albuminuria regardless of their diabetic status. Prior studies showed that ACEis and ARBs offer kidney protection by lowering proteinuria and slowing the rate of CKD progression [10]. Combination regimens with ACEis and ARBs are not recommended due to an increased risk of acute kidney injury and hyperkalemia.

Regarding the non-pharmacological therapies, KDIGO guidelines recommend the implementation of lifestyle modification among DKD patients, including low sodium intake (< 2 g/day), maintaining a protein intake of 0.8 g/kg/day for patients who are not on dialysis, and moderate-intensity physical activity for a cumulative duration of at least 150 min per week as tolerated [10].

## Data Availability

Not applicable.

## Abbreviations

CKD: Chronic kidney disease
eGFR: Estimated glomerular filtration rate
DKD: Diabetic kidney disease
ESKD: End-stage kidney disease
KDIGO: Kidney Disease: Improving Global Outcomes
ADA: American Diabetes Association
AKI: Acute kidney injury
AGE: Advanced glycation end-products
ACEi: Angiotensin-converting enzyme inhibitor
ARB: Angiotensin receptor blocker
